# Types of spectroscopy and microscopy techniques for cancer diagnosis: a review

**DOI:** 10.1007/s10103-022-03610-3

**Published:** 2022-07-14

**Authors:** Sindhoora Kaniyala Melanthota, Yury V. Kistenev, Ekaterina Borisova, Deyan Ivanov, Olga Zakharova, Andrey Boyko, Denis Vrazhnov, Dharshini Gopal, Shweta Chakrabarti, Shama Prasada K, Nirmal Mazumder

**Affiliations:** 1 Department of Biophysics, Manipal School of Life Sciences, Manipal Academy of Higher Education, Karnataka 576104 Manipal, India; 2 Laboratory of Biophotonics, Tomsk State University, Tomsk, 634050 Russia; 3 Central Research Laboratory, Siberian State Medical University, Tomsk, 634050 Russia; 4 Laboratory of Biophotonics, Institute of Electronics, Bulgarian Academy of Sciences, Tsarigradsko Chaussee Blvd, 72, 1784 Sofia, Bulgaria; 5 Biology Faculty, Saratov State University, 83, Astrakhanskaya Str, 410012 Saratov, Russia; 6 Department of Cell and Molecular Biology, Manipal School of Life Sciences, Manipal Academy of Higher Education, Karnataka 576104 Manipal, India

**Keywords:** Cancer, Fluorescence, Raman scattering, Nonlinear optical microscope, Photoacoustic signal

## Abstract

Cancer is a life-threatening disease that has claimed the lives of many people worldwide. With the current diagnostic methods, it is hard to determine cancer at an early stage, due to its versatile nature and lack of genomic biomarkers. The rapid development of biophotonics has emerged as a potential tool in cancer detection and diagnosis. Using the fluorescence, scattering, and absorption characteristics of cells and tissues, it is possible to detect cancer at an early stage. The diagnostic techniques addressed in this review are highly sensitive to the chemical and morphological changes in the cell and tissue during disease progression. These changes alter the fluorescence signal of the cell/tissue and are detected using spectroscopy and microscopy techniques including confocal and two-photon fluorescence (TPF). Further, second harmonic generation (SHG) microscopy reveals the morphological changes that occurred in non-centrosymmetric structures in the tissue, such as collagen. Again, Raman spectroscopy is a non-destructive method that provides a fingerprinting technique to differentiate benign and malignant tissue based on Raman signal. Photoacoustic microscopy and spectroscopy of tissue allow molecule-specific detection with high spatial resolution and penetration depth. In addition, terahertz spectroscopic studies reveal the variation of tissue water content during disease progression. In this review, we address the applications of spectroscopic and microscopic techniques for cancer detection based on the optical properties of the tissue. The discussed state-of-the-art techniques successfully determines malignancy to its rapid diagnosis.

## Introduction

Cancer is an uncontrolled growth of cells, invading nearby tissues via the blood and lymphatic system. It is the second leading cause of death, with 18 million new cases and 9.6 million deaths in 2018 [1]. In the same year, approximately 70% of cancer deaths are reported in low- and middle-income countries worldwide. Cancer development depends on biological factors (such as age, gender, and genetic inheritance), environmental factors (carcinogens, UV or radioactive radiation, and some chemicals), lifestyle, and health conditions (tobacco, alcohol, and presence of infections such as HIV and HPV). Detection of cancer at an early stage could be correlated with higher patient survival [2]. Breast cancer for women and lung as well as prostate cancer for men are the most frequently occurring cancers [3, 4]. Skin cancer is a significant problem for countries in hot climate zones due to high skin exposure to UV radiation. Melanoma is considered to be the most dangerous skin cancer. Still, basal cell carcinoma (BCC) and squamous cell carcinoma (SCC) are also very dangerous and require detection at an early stage for better clinical outcomes. Pathological examination of tissue samples is the standard diagnostic technique for cancer detection. However, this method involves removing tissue from suspected areas and identifying morphological abnormalities [5]. It is a time-consuming procedure and is entirely dependent on the judgment of the pathologist. The neoplastic tissue transformation is accompanied by change in tissue morphology and metabolism (see, for example, [6, 7]). The former can be discovered by visualization methods; the latter is associated with a variety of tissue chemical compositions, which can be detected by spectroscopy tools.

Sonography, mammography, positron emission tomography (PET), computed tomography, and magnetic resonance imaging (MRI) are the most commonly used methods for cancer visualization. The majority of them allow the detection of malignant tumors only at the stage of clinical manifestation. Magnetic resonance spectroscopy, magnetic resonance perfusion, diffusion-weighted, and diffusion tensor imaging are used to make accurate diagnosis and treatment decisions. However, these techniques are expensive, time-consuming, and require complex data processing.

Optical microscopy suitable for malignant tumor detection includes quantitative phase-contrast phase imaging [8, 9], optical coherence tomography [10], based on phase interference pattern registration, and polarization contrast microscopy based on control of optical wave polarization variations [11, 12]. Further, the differential interference contrast microscopy combines both polarization control and phase variation interferometry. This technique is suitable for operative segmentation of the lesion from normal tissue to determine the lesion border during cancer treatment by optical guided surgery. For differential diagnosis, it should be combined with the analysis of tissue chemical composition by spectral methods. Raman and fluorescence spectroscopies allow early-stage cancer diagnosis, by detecting circulating cancer cells or molecular cancer biomarkers in biofluids [13, 14].

In addition to these techniques, hyperspectral imaging (HSI) provides spatial and spectral information about the sample [15]. Along with ex vivo analysis, HSI is used to image solid tumors over a wide spectral range. The size and shape of the tumor are obtained by spatial information, whereas spectral information is used to determine the composition of the sample. Due to limitations with imaging depth, this technique can be easily applied in the detection of tumors in exterior organs such as skin [16, 17] and head [18]; however, deploying the same with endoscopic techniques enables the imaging of interior organs [19, 20]. Further, optical-based treatment techniques such as photodynamic therapy (PDT) also proved to be effective in cancer treatment. In PDT, the chemical agents known as photosensitizers or PDT drugs are activated upon illumination by light with a suitable wavelength, which in turn causes damage to the cancer cell by releasing oxygen radicals. This technique targets only the tumor cell without harming healthy cells. However, in some cases, the healthy cells near the tumor region could be minimally affected [21, 22]. This has proven to be effective in the treatment of skin cancer [23–25], lung cancer [26], head and neck cancer [27], etc.

In this review, we discuss fluorescence, Raman, photoacoustic, terahertz absorption spectroscopy, optical-based molecular imaging techniques in cancer studies, and their associated applications.

## Applications

### Fluorescence spectroscopy

The fluorescence phenomenon plays an important role in cancer diagnosis. It is based on the analysis of tissue emission spectra and is usually presented as a function of intensity vs. wavelength [28, 29]. Fluorescence spectroscopy can be performed through steady-state or time-resolved techniques, using external fluorescent protein labeling, single or multiple wavelength excitations, etc. [29–31]. Steady-state fluorescence is the most frequently used for emission spectral analysis. The autofluorescence is achieved without introducing external fluorescence labels, which allows in vivo detection without tissue sample preparation [32]. The measurement modality and the parameters of the fluorescent signal to be studied are decided depending on the clinical task such as establishing a primary diagnosis for an unknown lesion or monitoring its development [30, 31], to evaluate tumor treatment [33] or guide its surgical excision [34, 35]. The success of fluorescence spectroscopy for tumor detection depends on the lesion’s morphological structure, biochemical content, and metabolic status. When working in vivo, the tissue pH, temperature, and ionic balance in the cells are strongly influenced by the fluorescence signal. The tissue pH can be determined using a pH-sensitive fluorophore. For example, the serotonin emission maximum is shifted from 330 to 550 nm without a change in its absorption spectrum when the pH changes from neutral to strongly acidic (pH decreases) [36]. A more acidic environment of a tumor also influences the fluorescence of pH-sensitive molecules, which could be used as a cancer biomarker.

Hypoxia is typical for solid tumors, and it affects cellular respiration, which is reflected by a change in the ratio of major fluorescent coenzymes in the tissues such as nicotine adenine dinucleotide (NAD) and flavin adenine dinucleotide (FAD). This alteration can be traced by measuring the intensity of their fluorescence maxima or fluorescence lifetime [37–39]. Pathological changes in cell metabolism are evaluated by the fluorescence signal of amino acids such as tyrosine and tryptophan. Due to the intensive metabolism rate in tumor cells and their abnormal proliferation rate, a higher uptake of tryptophan from the microenvironment is required. On the other hand, tryptophan is sensitive to the environment’s acidity, and hence, its fluorescence emission maximum shifts to longer wavelengths as the tissue pH level decreases [40, 41]. Abnormal tumor growth and secreting metalloproteinases (a kind of enzymes) destroy the tissue’s extracellular matrix and reduce the content of structural proteins in the tumor area [42]. As a result, low-intensity fluorescence maxima from collagen and elastin are observed in tumors when compared to healthy tissues.

**Fig. 1:**
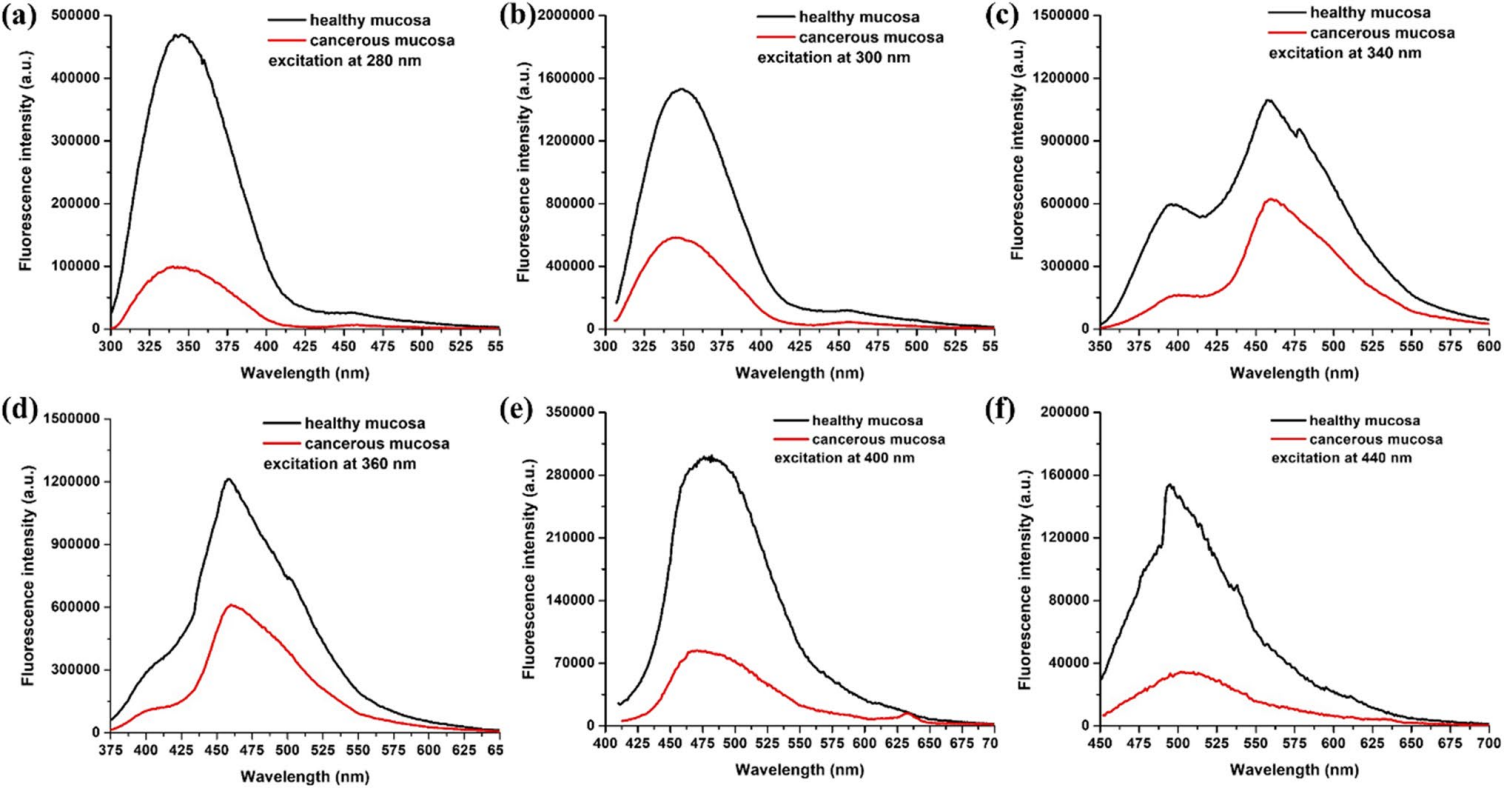
Autofluorescence spectra of normal and cancerous colon mucosa using different excitation wavelengths: **a** 280 nm; **b** 300 nm; **c** 340 nm; **d** 360 nm; **e** 400 nm; and **f** 440 nm

Probably, one of the earliest light-induced fluorescence (LIF)-identified indicators of cancer was a variation of endogenous porphyrin fluorescence [43–46]. The accumulation of porphyrins in the tissue is an indicator of pathological processes such as bacterial infections, inflammation, as well as oncological transformation [32, 47–49]. Figure 1 presents the autofluorescence spectra of normal mucosa and carcinoma of the lower gastrointestinal tract for a set of excitation wavelengths, which demonstrates the difference in endogenous fluorophore emission between healthy and diseased tissues. Thus, the ratios between different emission maxima and even the appearance of a new one (in the red spectral region) correspond to the accumulation of an endogenous porphyrin in the tumor. Additionally, steady-state fluorescence intensity decreased in cancerous tissues. Further, laser-induced autofluorescence (LIAF) lifetime spectroscopy is helpful to differentiate between normal and cancer tissues in squamous cell carcinoma (SCC) [14, 50], cervical cancer [35], and brain tumors [51–55]. The mean fluorescence lifetime was established to be 3.75 ± 0.49 ns for normal tissues and 4.37 ± 0.85 ns for malignant tissues [14] providing an evident distinction between normal and tumor tissues [50, 56–58].

Significant changes in tissue morphological and biochemical characteristics are observed in the process of malignant lesion development [46, 58, 59]. It is a reason for the essential variances in the fluorescence spectra of normal and cancer tissues, which can be used as diagnostic biomarkers and/or predictors for tumor lesion development. Fluorescence spectroscopy was applied for tumor detection in different organs and tissues, including lung [60], brain [34], skin [32, 33, 47]. gastrointestinal tract [30, 42], liver [39], cervix [61], etc.

### Fluorescence microscopy

Confocal fluorescence microscopy (CFM) and TPF microscopy are optical methods allowing non-invasive imaging in vivo of untreated biological tissues at a cellular-level resolution. CFM provides high-resolution three-dimensional (3D) visualization by suppressing any signal out of the focus plane using a pinhole in front of the detector. The variation in the pinhole aperture size facilitates the selective focal plane imaging of the sample [62–65]. CFM allows the acquisition of an autofluorescence image with higher lateral and axial resolution compared to wide-field fluorescence spectroscopy. CFM is performed both ex vivo [66] on untreated or treated tissue samples and in vivo [67, 68] as a gastrointestinal tract micro-endoscopy technique.

The most popular wavelength used for excitation in CFM is 488 nm. When such excitation is applied, the fluorescence signal is expected to be mainly due to the structural proteins such as collagen and elastin in the study of ex vivo samples, and in vivo, the coenzymes NAD and FAD may also contribute to the fluorescence signal [66, 67]. This method is already being applied in clinical practice [69]. CFM has several disadvantages, such as intense photobleaching and loss of the fluorescence signal which can be avoided by applying TPF, characterized by more efficient penetration of the excitation light into biological tissue [70, 71]. TPF is based on two photons’ simultaneous absorption, a process requiring a high photon flux in the illuminated tissue’s excitation volume. This condition is achieved with the use of ultrashort pulse lasers. In TPF, the excitation and fluorescence processes are most likely in the laser beam’s focal plane. The resulting image is characterized by a lateral resolution comparable to that of CFM images, however without photobleaching of fluorophores in the sample. The simultaneous interaction of two photons with a molecule requires radiation with a wavelength twice higher in value than the studied tissue fluorophore’s excitation wavelength. Most of the diagnostically significant fluorophores are excited in the spectral range of 280–500 nm under CFM, and the TPF wavelengths have to fall into the therapeutic window which has a spectral range between 600 and 1000 nm. The absorption is low in this range allowing light to penetrate the tissue up to several centimeters [72]. It enables the study of larger volumes of tissue with deeply situated objects of interest, such as neoplastic formation and other pathologic disorders compared to CFM.

**Fig. 2:**
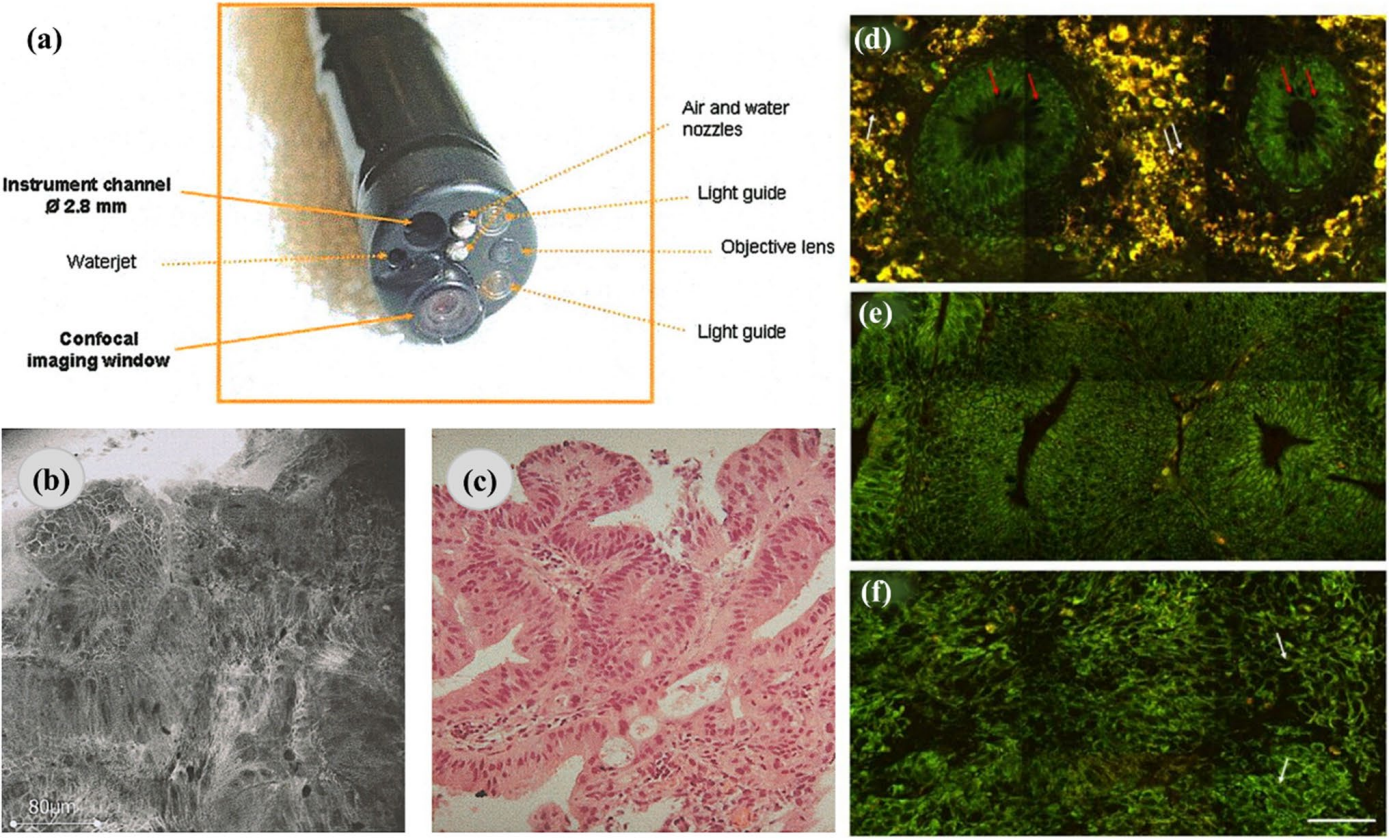
The application of confocal and multiphoton microscopy in cancer diagnosis. **a** A distal end of the confocal endomicroscopy. **b**, **c** Irregular cell architecture with a total loss of goblet cells and corresponding histologic specimen, respectively. Representative multiphoton images from normal (**d**), precancerous (**e**), and cancerous (**f**) colonic tissues at a depth of 0 μm. The excitation wavelength, *λ*_ex_, was 800 nm. Scale bar = 50 μm. The figures are reproduced with the kind permission from [70, 73]

A confocal endoscopy system was used for imaging colon tissues and predicting histology for colorectal cancer during the colonoscopy (as shown in Fig. 2). Blue laser light along with acriflavine hydrochloride and fluorescein sodium was used in producing high-quality deep images of the lamina propria. In the study, using fluorescein sodium, around 13,020 confocal images were obtained and compared with the histology analysis of 1038 biopsy specimens. The neoplasm was detected with 97.4% sensitivity, 99.4% specificity, and 99.2% accuracy. This diagnostic tool could have great potential in detecting neoplastic changes during colonoscopy [73]. Clark et al. [74] demonstrated the feature detection of neoplastic oral mucosa by reflectance confocal microscopy (RCM). RCM images of oral squamous cell carcinoma allowed the authors to determine tissue features providing a higher image contrast compared to histological images. The different nuclear densities of oral neoplastic and non-neoplastic tissues were established. Features like fibrosis, inflammation, muscle fibers, and salivary glands were also determined in the RCM images. Hence, confocal imaging could track the response to treatment and real-time detection of tumor margins by evaluating oral lesions [74]. Segura et al. [75] also used RCM to differentiate between lesions associated with melanoma and non-melanoma of the epidermal layer [75]. A two-step algorithm was developed to diagnose skin tumors with the help of RCM. It could identify a typical nucleated cells and round suprabasal cells, which were melanocytic and nevi typical basal cells. The morphological structures and skin features were detected in the fluorescence images, which correlated well with the histological analysis. Images obtained from multi-spectral RCM provided tissue spectral responses and complimentary fluorescence images allowing to differentiate the normal and cancerous structures [76].

A two-photon optical fiber fluorescence probe was employed for both in vivo and ex vivo studies of xenograft tumors using green fluorescence protein (GFP) [77]. An exponential increase in the fluorescent signal was obtained with an increasing amount of GFP-expressing cells. The probe was also used to detect and identify a Herceptin antibody targeted to Herceptin-2-expressing tumors in mice with severe combined immunodeficiency (SCID) [77]. A folic acid targeted to dendrimer nanoparticles and 6-TAMRA (6-carboxytetramethylrhodamine) was used as a fluorescent probe to tag KB cell tumors. A fourfold fluorescence signal increase in the tumor was obtained via the targeted dendrimer. A study conducted by Allocca et al. presented bone colonization using confocal and TPF microscopes [78]. Different protocols for bone cancer metastasis in a mouse model are discussed, suggesting that confocal/TPF microscopes could be a powerful tool for investigating and understanding microscale cellular structures and interactions. Two-photon autofluorescence microscopy is also used for imaging 150 μm below the epithelial surface. The image contrast was found to degrade below a depth of 320 μm due to strong scattering and low staining inhomogeneity of the microscope. However, the imaging depth could be improved using temporal focusing, spatial filtering, optical clearing, and differential aberration imaging. Overall, more development and advances in TPF microscopies could be more useful for in vivo optical biopsies [79]. Therefore, confocal and TPF microscopy approaches play a crucial role in studying, analyzing, and interpreting the tumor and have a prominent cancer diagnosis role. Table 1 summarizes the significance of the technique in the detection of various types of cancers.

**Table 1:** Principle and significance of various microscopic and spectroscopic techniques in cancer studies

Technique	Instrumentation	Information	Significance in cancer research	Reference
**Fluorescence spectroscopy**	Measures the fluorescence emission signal from the sample, when it is excited by a specific wavelength of light	Rapid detection of fluorescence and auto-fluorescence intensities Limit of detection ~ ng/mL or ppm	Spectral profiles of NADH, FAD, and tryptophan can be analyzed to differentiate between normal and cancerous tissues Biomolecules autofluorescence can be estimated to understand the lesion’s morphological structure and biochemical content, metabolic status	[32, 38, 39, 47]
**Fluorescence microscopy**	Fluorophores are used to tag the samples and are excited by a specific wavelength of light to visualize the sample	Depth of imaging ~ 320 μm 3D imaging capability Higher depth penetration of better imaging	Visualize the metabolism and biochemical changes of the cells which change during the progression of cancer Observe the changes in nuclear densities and cellular structures to distinguish cancer tissues Autofluorescence imaging for level free detection NAD and FAD to observe structural collagen and elastin changes in tissues	[70, 74, 78, 79]
**Second-harmonic generation microscopy**		Label-free imaging technique with reduced photodamage and photobleaching Deep tissue imaging up to several hundred microns 3D imaging capability Imaging depth ~ 1 mm	Label-free visualization of cancer tissues without phototoxicity to the samples Incorporation of polarization property with the SHG provides information about tissue ultrastructural changes Observe the changes in biomolecules levels and mitochondrial energy metabolism to detect pathological states	[80–84]
**Raman spectroscopy**	Measure the vibrational mode densities of the different bonds present in the sample to classify them	Label-free detection of vibrational bands Limit of detection ~ ng/mL or ppm Spectral resolution ~ 40 μm	Identifies specific chemicals generated during malignancies Help in distinguishing tissues based on metabolites levels Can detect lipid-rich structures, which makes it ideal for use in studies involving the measurement of various cellular contents	[85–89]
**Photoacoustic spectroscopy**	Measures the consequent generation of an acoustic wave due to absorption of optical radiation with amplitude modulation at a few Hz up to several kHz frequencies by the sample	Level of detection ~ 100 ppm	Detection of volatile biomarkers which are associated with early diagnosis of cancer Distinguish between normal and malignant tissues based on structural changes A non-invasive method to monitor the progression of cancer	[90–93]
**Photoacoustic microscopy and tomography**	Provide visualization of the different anomalies in the sample due to generation of an acoustic wave from to absorption of optical radiation	Depth of imaging ~ cm Lateral resolution − 50 to 100 μm Axial resolution ~ 20 μm	Label-free analysis of tissues using endogenous chromophores Determination of different oxygen level regions among tissues to distinguish overgrowth Provides better contrast in the determination of metastasis based on blood flow profiles and vasculature	[94–98]
**Terahertz spectroscopy**	Uses terahertz waves to detect hydrogen bond of water to classify samples based on their water contents	Label of detection ~ 10 µmol/L	Rapid determination of liquid biomarkers for early detection of cancer Easy micro-RNA and exosomes detection in biofluids as a detection marker of cancer Distinguish biochemical properties of blood serum between healthy and diseased individual	[99–104]

### Second-harmonic generation microscopy

In conventional optical microscopic techniques, the optical response of the sample is linearly dependent on the intensity of the incident light. In contrast, in nonlinear methods such as second-harmonic generation (SHG) microscopy, the SHG signal is produced by multiple photons’ interaction with biological samples mainly from the non-centrosymmetric molecules. This nonlinear technique provides low photon toxicity enabling 3D imaging of tissue and organ structures [80, 105]. In the extracellular matrix (ECM), collagen is the major constituent capable of generating SHG signals. During cancer progression, collagen undergoes specific morphological changes, and its SHG signal is useful in the diagnostics and prognostics of cancer [81, 82]. It is crucial to assess the metastatic potential in lymph node-negative (LNN), estrogen receptor-positive (ER +) cancer patients to avoid over-treatment. Burke et al. predicted the metastasis in cancer patients based on the forward-to-backward emitted SHG signal ratio from fibrillar collagen. The outcome of the developed matrix-based approach complemented traditional genomic cell-based methods. Hence, it can be used as a prognostic indicator of metastasis risk [106, 107].

**Fig. 3:**
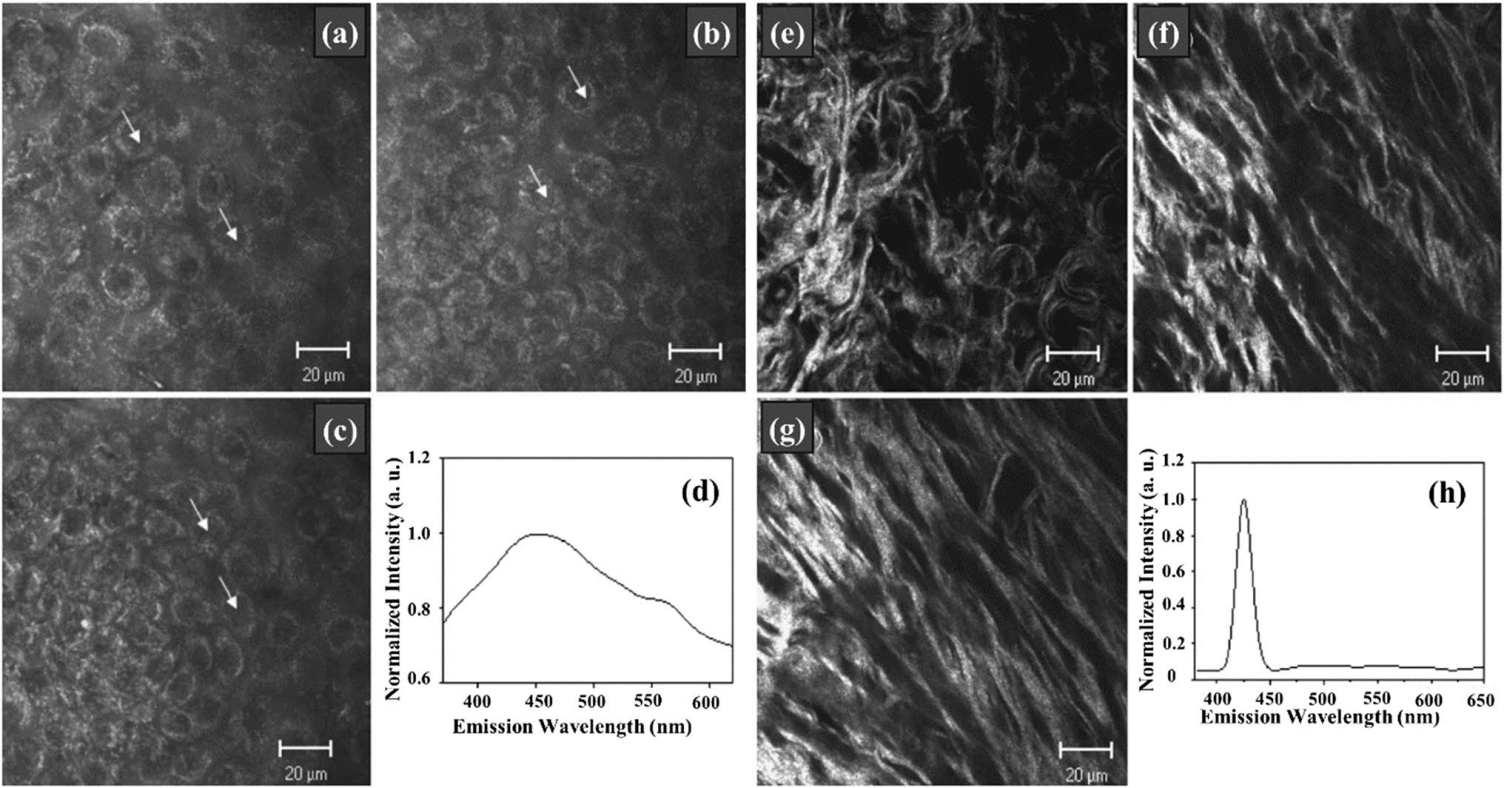
**a**–**c** Multiphoton images based on TPF of the cervical epithelium at depths of 5 μm, 10 μm, and 15 μm. **d** Corresponding spectrum of the epithelium. The excitation wavelength, *λ*_ex_, was 760 nm. The scale bar represents 20 μm. **e**–**g** Multiphoton images based on the SHG of cervical stroma at depths of 40 μm, 50 μm, and 80 μm. **h** Corresponding spectrum of the stroma. The scale bar represents 20 μm (*λ*_ex_ = 850 nm). The figure is reproduced with the kind permission from [84]

Again, polarization-resolved SHG microscopy is a potential tool in cancer diagnostics, providing quantitative information regarding the tissue’s ultrastructural changes. Double Stokes-Mueller polarimetric SHG microscope-based imaging is employed to study the correlation of various polarization parameters between normal and breast cancer tissues. The study is carried out with linear incoming and outgoing polarization states of the SHG signal. The nonlinear susceptibility component ratio (*R*), average in-plane orientation, and degree of linear polarization (DLP) are used to quantify the tissue malignancy. It is observed that the collagen orientation increases during cancer progression, which in turn causes variation in the *R* and DLP of the sample. Hence, measurement of SHG polarimetry along with SHG intensity helps to provide a detailed understanding of tissue collagen as a cancer biomarker [83, 108]. In a study conducted by Zhuo et al. [84], multiphoton microscopy was used to visualize the cervical tissue structure to detect the malignancy in the early stages (as shown in Fig. 3). The cervical tissue of the mouse was used to image epithelium cells along with the underlying stroma by TPF (*λ*_ex_ = 760 nm) and SHG (*λ*_ex_ = 850 nm) techniques. This study revealed a two-layered structure of the epithelium cell, and it also aided in the structural analysis of the same. The ratio of nicotinamide adenine dinucleotide (NADH) over flavoproteins (FAD) co-enzyme provides information regarding mitochondrial energy metabolism, which can detect the pathological changes between normal and malignant tissues [84].

An endoscopic tool capable of acquiring high-resolution SHG images of cervical collagen to remodel abnormalities related to preterm birth was created [109]. The systematic study using murine cervical tissue provided information regarding the progressive changes in collagen content and orientation during pregnancy. This non-invasive technique showed convincing results over the benchtop SHG tool in clinical assessment. SHG microscopic technique was used to study the orientation of collagen in the cervix tissue, which helps in cervical remodeling to investigate menopausal intricacies. In the study, an increased collagen fiber organization was observed in post-menopausal women compared to their pre-menopausal examination [110]. The SHG imaging technique was also conducted simultaneously with fluorescence lifetime imaging to study the cancer indicators and monitor the effect of different chemotherapeutic drugs in colon cancer (CT26)-induced mouse models [111].

### Raman spectroscopy

Raman scattering allows the study of structure and binding of molecules by examination of their scattering properties. It has managed to gain considerable attention, as it is a non-destructive technique and provides precise information at a molecular level [112]. This technique is easy to use as it does not need any sample preparation; thus, it can be employed in vivo. Raman spectrum contains information about the vibrational mode density, which is then converted to chemical composition [113]. Based on the Raman fingerprint, it is possible to differentiate between benign and malignant tissues in various cancer types [85, 86].

A challenge in detecting skin cancer is to distinguish cancer tissue from the surrounding non-cancerous tissue. Confocal Raman spectroscopy with 514.5-nm excitation wavelength distinguishes BCC from normal tissues. Raman depth profiling also accurately separated the healthy surrounding tissue from BCC. Tissue sections were used for both confocal Raman spectroscopy profiling and H&E staining. H&E-stained images have shown dark and lighter regions that are BCC and non-cancerous tissues, respectively. Distinct Raman spectral differences for the amide I mode and PO-2 symmetric stretching mode between normal and BCC tissues showed its diagnostic potential [114]. A confocal Raman microscopy was used for lung cancer detection. The stained sections of normal lung tissue were used as a reference and scanned with a microscope. Data reduction by principal component analysis (PCA) was used in combination with Raman microscopy to analyze the variation between the spectra and group them using similarities. PCA with Raman microscopy distinguished healthy and malignant lung tissues with an 84% sensitivity and 61% specificity. Many subtle differences in intensity between the two main spectra can be identified along with the main differences in the intensity of amide I band in lung tumors. Raman microscopy can differentiate between malignant and normal bronchial tissues and predict the postoperative occurrence of cancer [87].

**Fig. 4:**
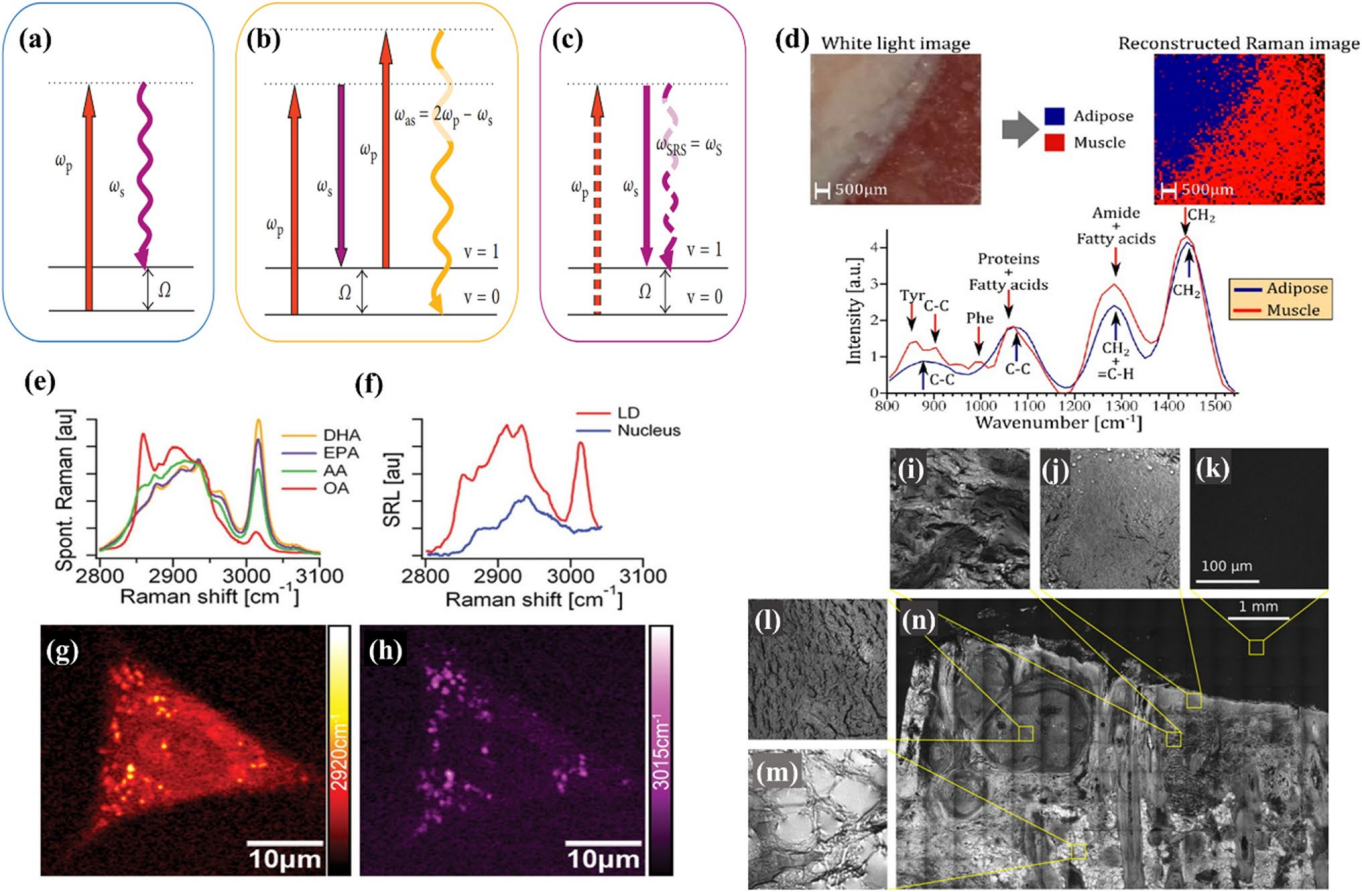
Energy diagrams of **a** spontaneous Raman scattering, **b** CARS, and **c** SRS. **d** White light photograph of the region imaged with the wide-field system with a respective false-color rendering of the classification result and representative normalized spectra acquired with the wide-field system for adipose and muscle tissue. Omega-3 fatty acid uptake by A549 human lung cancer cells was monitored with SRL microscopy and microspectroscopy. **e** Spontaneous Raman spectra of docosahexaenoic acid (DHA, with six C = C bonds), eicosapentaenoic acid (EPA, with five C = C bonds), arachidonic acid (AA, with four C = C bonds), and oleic acid (OA, with a single C = C bond). The strong Raman peak around 3015 cm^–1^ is characteristic of unsaturated fatty acids. **f** SRL spectra of a lipid droplet (LD, red line) and a region inside the nucleus (blue line). Unlike the nuclear region, the SRL spectrum of the LD shows good correspondence with the spectra of pure EPA shown in (a). **g** SRL image of a cell at 2920 cm^–1^. **h** SRL image of the same cell at 3015 cm.^–1^. These findings indicate that EPA is taken up by the cells and is more strongly enriched in LDs compared to other cellular organelles. CARS image of a skin section with BCC with various texture regions highlighted: **i** skin dermis, **j** stratum granulosum, **k** image background, **l** tumor region, **m** adipose tissue, and **n** overview of the sample. The figures are reproduced with the kind permission from [115–118]

Coherent anti-stokes Raman scattering (CARS) and stimulated Raman scattering (SRS) are Raman processes providing high sensitivity, contrast, and operativeness (as shown in Fig. 4). SRS is a third-order nonlinear optical process that uses a narrow-band ultrashort pulse laser. The incident photons excit the molecule to vibration transitions and cause stimulated Raman gain or loss of photons.

Improving chemical specificity to identify various molecular components in biological samples raises the need to enhance the imaging selectivity, which can be done by integrating single-frequency SRS imaging with confocal Raman spectroscopy [115, 119]. The spectral range between 300 and 1800 cm^−1^ belongs to the vibrational modes of DNA, proteins, and lipids, whereas a CH_2_ stretch in the 2850 cm^−1^ region is associated with lipid content. Taking this into account, Raman spectroscopy efficiently distinguishes healthy and cancerous cells [120]. Hyperspectral SRS imaging of tissue revealed anomalous accumulation of saturated fat in the cancerous tissue. The liver malignant tumor along with surrounding benign tissues was analyzed by hyperspectral SRS microscopy. A significant expansion of the two types of fats was observed in the cancerous liver tissue. One of them formed irregular large droplets that had a Raman band peak at 2885 cm^−1^, which is a key feature of saturated lipids. The other formed intracellular lipid droplets that had distinct Raman bands at 3007 cm^−1^ corresponding to unsaturated lipids. In contrast, the adjacent normal liver tissues had a little saturated fat along with well-organized cellular morphology. With the capability of chemical content analysis and high spatial resolution, SRS can readily determine in situ metabolites in unprocessed cancerous tissue, which provides a great insight into the clinical applications for cancer diagnosis [121].

Similar to SRS, CARS microscopy combines chemical content analysis with high-resolution 3D label-free imaging capability. A CARS microscopy setup includes two lasers, coherently exciting a Raman vibration mode. For example, the vibrational mode at 2850 cm^−1^ corresponds to CH_2_ stretch and is specific for lipids. Therefore, the CARS signal can detect lipid-rich structures, which makes it ideal for cellular content studies [88, 122]. Excess lipid content was established in hormone-responsive breast and prostate cancer treated with the hormone medroxyprogesterone acetate and R1881, respectively. CARS microscopy was used to characterize the increase in the quantity and size of lipid droplets in the cells [89].

### Photoacoustic spectroscopy

Photoacoustic spectroscopy (PAS) uses the absorption of optical radiation with amplitude modulation at a few Hz up to several kHz frequencies by a molecule and consequent generation of an acoustic wave. If the period of repetition of a laser beam amplitude variation exceeds the non-radiative relaxation time, then the acoustic wave with the same frequency is generated and can be registered by a microphone. When the laser beam of a particular wavelength is scanned over a spectral interval, the microphone signal measures an absorption spectrum of a gas sample in the cell [90–123]. A mechanical chopping of a continuous wave (CW) laser radiation [124] or nanosecond pulse duration laser can be used to provide the necessary amplitude modulation to the gas sample. The construction of a photoacoustic detector (PAD) cell influences the sensitivity of PAS [125].

**Fig. 5:**
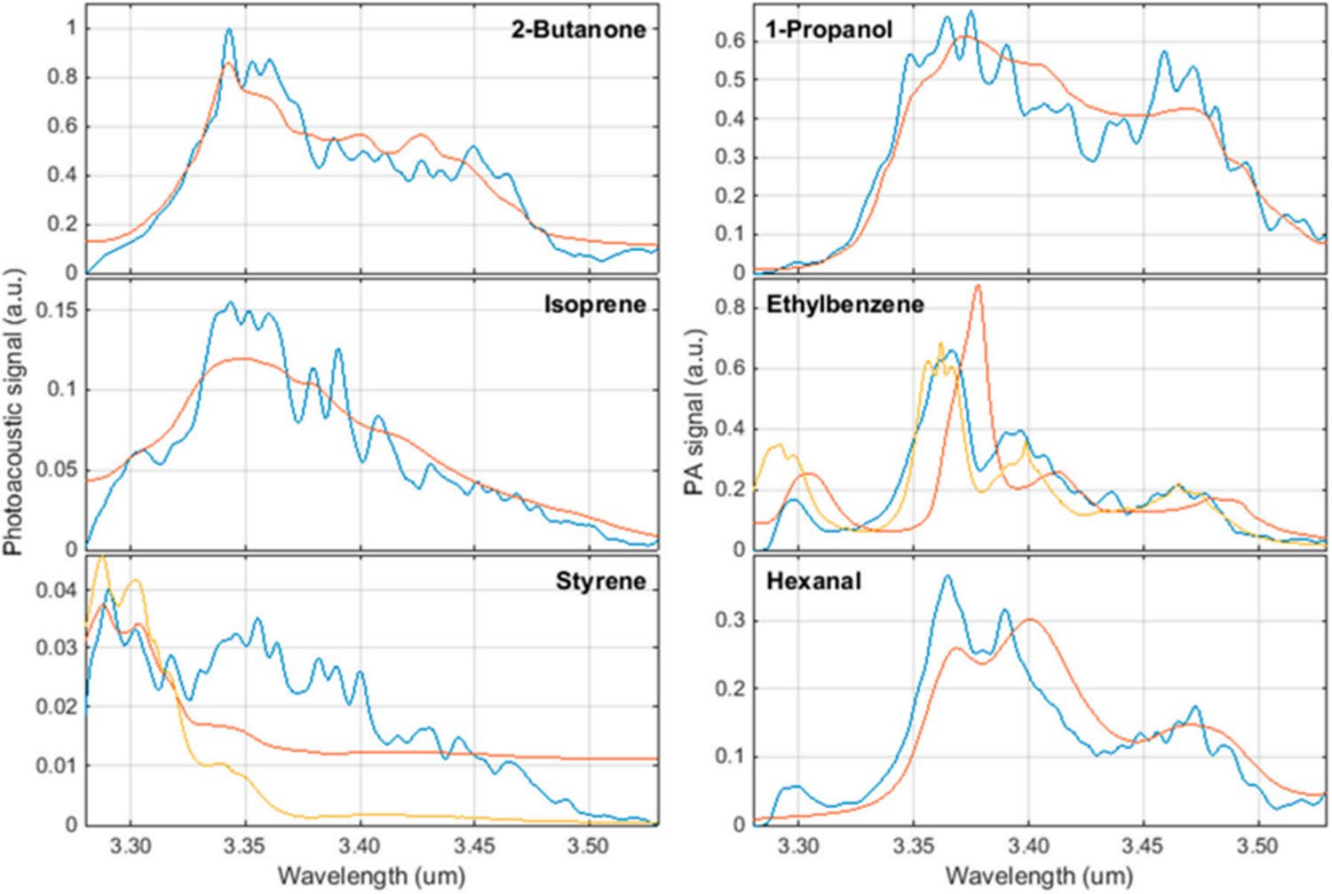
Biomarker spectra (blue: measurement; red: NIST; yellow: PNNL) was measured at a concentration of 100 ppm in nitrogen at atmospheric conditions (294 K, 1024 hPa) [92]

Optical parametric oscillators (OPOs) are the most widespread laser sources generating coherent radiation tunable in a wide spectral range. The OPO-based PAS device with an average resolution of 0.18 nm, tunable in the range of 3270–3530 nm for propane, ethane, and methane with a detection limit of 100 ppm, was demonstrated [125]. The average relative error between the measured spectral and the corresponding normalized intensities from the HITRAN database was 7.1–6% for propane and 15–14% for methane [125]. A PAS sensor for detecting volatile organic compounds (VOCs) relevant to lung cancer has been studied. The following substances were studied as biomarkers of lung cancer: isoprene, styrene, 1-propanol, 2-butanone, ethylbenzene, and hexanal. In a study, an OPO with a tuning range from 3.2 µm to 3.5 µm was used as a laser source with a detection limit varying from 5 to 142 ppb for the VOCs as mentioned [92]. The experimental results were compared with reference data from the National Institute of Standards and Technology (NIST) database. The observed spectra of ethylene, benzene, and hexane shifted by approx. 20 nm towards shorter wavelengths compared to the reference spectra of NIST (Fig. 5). The experimentally obtained spectrum of styrene differed significantly from the reference one.

In a study, pulmonary disease was detected using PAS spectral analysis of breath air by LaserBreeze gas analyzer. This study included 18 lung cancer patients, 21 patients with pneumonia, 22 patients with chronic obstructive pulmonary disease, and 39 healthy nonsmoking volunteers [125]. The PAS LaserBreeze gas analyzer uses a wavelength range of 2.5–10.7 μm [126], and the laser source includes two automatically switched OPOs pumped by Nd:YLF laser. The experimental data analysis was carried out using a support vector machine (SVM) which is a differential classification predictive machine learning. Informative feature extraction was realized by PCA, and the model provided up to 90% binary classification accuracy [125, 127, 128]. The PAS was used to monitor breast cancer development [129] with frequency-doubled 281-nm excitation source using an Nd:YAG and dye laser. A suitable number of MCF-7 cells were injected in female nude mice to induce tumor and developed for 20 days. The tumors were analyzed at 10, 15, and 20 days after tumor inoculation. The wavelet-PCA-based logistic regression of data identified unique photoacoustic spectral patterns among the group of samples leading to the differentiated tumor progression. The most specific frequencies were 5.93 kHz, 15.9 kHz, 29.69 kHz, and 32.5 kHz. The analysis prediction accuracy for the 10th day versus 15th day, day 15th versus 20th day, and 10th day versus 20th day were found to be 92.31, 87.5, and 95.2%, respectively.

The PAS ovarian tissue study and the subsequent statistical analysis are performed using PCA, kNN, ANN, and SVM algorithms [130]. ANN and k-NN algorithms demonstrated relative accuracy with 100% specificity and 86.76% sensitivity, whereas SVM showed 100% and 80.18% of sensitivity and specificity, respectively. The PAS was applied for distinguishing breast cancer patients from healthy controls using blood serum analysis [93]. Three time-domain photoacoustic spectra were recorded for each of 20 normal and 20 malignant samples with 281-nm pulsed laser excitation. In total, 120 spectra were recorded by a combination of wavelet analysis, PCA, and logistic regression that was applied in the selected frequency regions of 0–100 kHz and 116.56–206.87 kHz. In common, the limitations of PAS are as follows; the detection limit depends on the power of laser radiation source, and high-power laser sources are preferable. The PAS requires normal pressure for collisional relaxation and generating intensive acoustic waves. Thus, the sensitivity is too low at pressures much below 1 bar. PAS equipment calibration is necessary to measure absolute concentrations as well [131].

### Photoacoustic microscopy and tomography

Photoacoustic microscopy and tomography applied in both single-cell, tissue, in vitro, and in vivo imaging demonstrate a significant growth over the past decade [94]. Based on the optical excitation and acoustic detection modality, the photoacoustic microscopy and tomography consist of three experimental techniques, involving photoacoustic computed tomography (PACT), acoustic-resolution photoacoustic microscopy (AR-PAM), and optical-resolution photoacoustic microscopy (OR-PAM). The optical beam focusing defines the penetration and spatial resolution of photoacoustic microscopy (PAM). Reducing the imaging depth improves spatial resolution and vice versa. Typically, the imaging depth ratio with PAM’s spatial resolution is ~ 200 [132]. The axial resolution also depends on a transducer’s geometrical focus and bandwidth [94]. PACT realizes wide-field optical microscopy with micrometer spatial resolution providing high penetration up to several centimeters. The OR-PAM differs from AR-PAM based on optical beam and acoustic wave focusing [95]. In OR-PAM, the optical focus spot is much smaller than the acoustic focus, and in AR-PAM, it is opposite. In both cases, the axial resolution is determined acoustically. The OR-PAM has a finer lateral resolution whereas AR-PAM has a finer axial resolution [132, 133]. The AR-PAM uses either tightly/loosely focused optical illumination or focused acoustic detection to provide high spatial resolution with relatively less penetration depth [95]. PAM’s primary implementation schemes are transmission mode, where the optical pump source and ultrasonic detector are on opposite sides of the sample, and reflection mode, where they are on the same side [94]. The latter is more suitable for in vivo measurements.

**Fig. 6:**
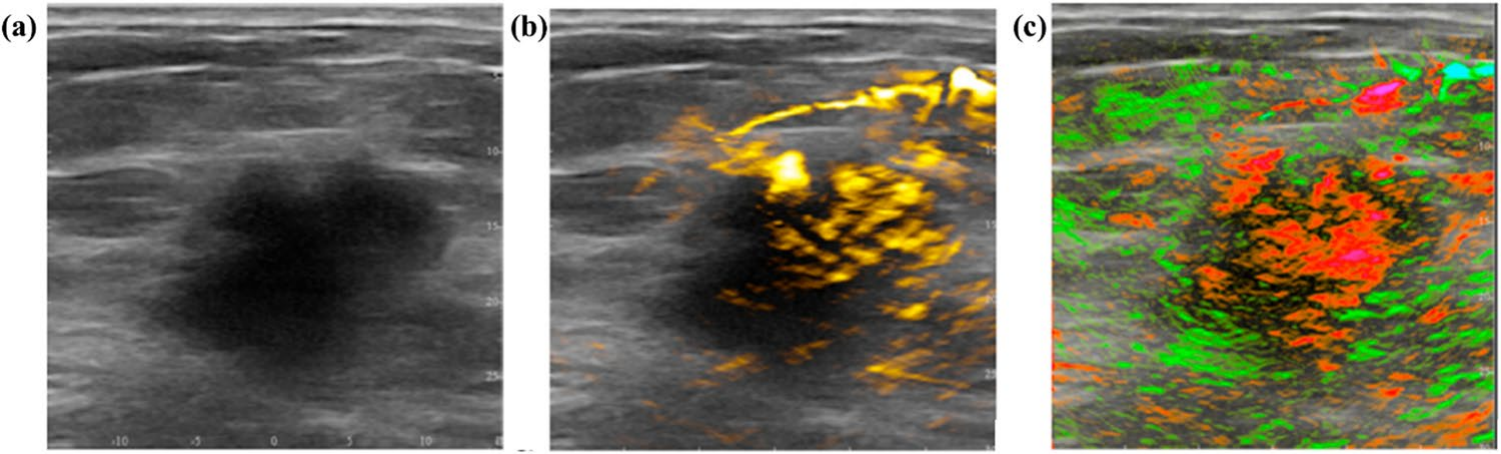
The example of a breast carcinoma OA/US imaging: **a** a 2.6-cm malignant mass on grayscale ultrasound; **b** the overlaying US with OA imaging, which illuminates increased internal total hemoglobin; **c** the overlaying US with OA imaging, which illuminates diffuse internal blood deoxygenation. The figure is reproduced with the kind permission from [96]

In common, the detection limit of PAM is determined by the laser radiation wavelength, intensity, imaging depth, tissue absorption, and ultrasonic transducer efficiency [133]. One of PAM’s principle benefits is the ability to provide label-free analysis of tissues using endogenous chromophores, such as hemoglobin in red blood cells, cytochromes in mitochondria, melanin in melanosomes, DNA, and RNA within the cell nucleus [133]. A specific chromophore control in tissue can be provided by adjusting the excitation wavelength to its absorption peaks. For example, blood oxygen saturation can be quantified by measuring the spectral differences between oxyhemoglobin and deoxyhemoglobin with high efficiency compared to other tumor hypoxia imaging methods (e.g., blood oxygen level-dependent MRI and PET) [134, 135]. Acute lymphoblastic leukemia causes hypoxic regions in the bone marrow. The corresponding local intravascular oxygen saturation can be detected by PAM [136]. The combined PAM/Ultra sound (US) system for breast morphology imaging, including cancer, is based upon the grayscale US combined with PAM, allowing the control of total hemoglobin and blood oxygen saturation [96]. An example of breast carcinoma PAM/US imaging is presented in Fig. 6.

The fundamental vibrational absorption levels have been used for PAM detection of glucose in tissues [137]. Thus, vibration-based PAM is a promising base for molecular-specific tissue imaging applications. The main drawback of PAM is the masking effect produced by the absorption of water in tissue. The reduction of the latter can occur in two ways. One is through the stimulated Raman process, and the other is through an overtone transition [138]. Photoacoustic flow cytometry (PAFC) is also another high sensitive method for cell or biological nanoparticles analysis in blood flow [139]. PAFC is based on laser pulses interaction with the blood in small vessels placed close to the body surface and the consequent recording of the induced acoustic waves by an ultrasound detector and provides a single-cell sensitivity level. PAFC has been used for label-free detection of circulating tumor cell (CTC) in vivo in the bloodstream, with the sensitivity being 100-fold higher than in vitro laboratory methods [140–142]. Blood flow can be analyzed with perfusion imaging or flow imaging. The latter allows the mapping of functional vascularity or the flow profile in an artery or vein. Imaging vascularity allows the identification of angiogenesis when the new vasculature’s fast growth occurs [143]. Photoacoustic imaging enables both, however, the latter is of primary interest because the malignant tissue is unorganized and has dense vasculature compared to normal tissue. The high density of the blood vessel results in an increased PAM contrast, enabling tumor location [135]. Photoacoustic angiography has been developed, allowing vascular shape quantification and density analysis. The observed diameter of blood vessels did not exceed 100 μm [97]. An OR-PAM technique tracks vascular changes in a mouse model of prostate cancer treatment using a DC101 anti-angiogenic agent. Approximately 1–3 days after the initial therapy, OR-PAM detected tumor vessel tortuosity reduction, vessel diameter decreasing, and intratumoral vessel distribution homogenization [144]. Thus, PAM allows the detection of optical absorbers in the tissue. This technique is attractive for clinical applications due to its high resolution and suitable penetration depth. The possible applications are functional brain imaging, breast cancer screening, psoriasis, skin lesion diagnosis, surgery and tumor therapy control, and lymph node tumor metastasis imaging [98].

### Terahertz spectroscopy

Terahertz (THz) waves are highly sensitive to the presence of water due to the strong absorption of hydrogen bonds in the THz region. This property can be used to detect cancerous areas where the water content is higher than in normal tissue because of their excessive metabolism [99, 100]. The disadvantage of this approach is that water molecules cause significant attenuation of THz waves [145]. Tissue freezing, paraffin embedding, vaporization [146]. and penetration-enhancing agents were used to reduce water absorption [147]. Despite the problems mentioned above, several successful studies of biofluids such as blood, saliva, and urine by THz spectroscopy were carried out.

**Fig. 7:**
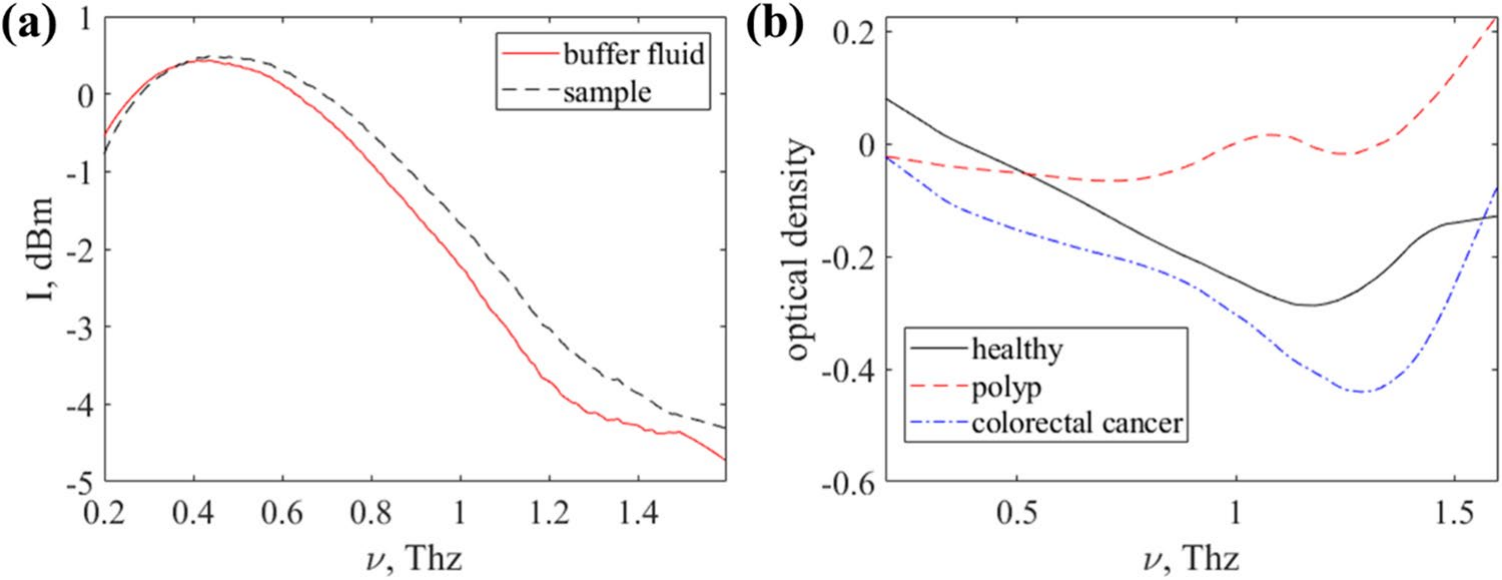
**a** Intensity THz spectra of a buffer liquid and a sample with exosomes and **b** example of optical density spectra obtained using attenuated total reflection mode with a fluoroplastic prism for a healthy donor and patients with polyps and colorectal cancer

Saliva contains sialic acid, which is known as an early indicator of breast cancer [148]. The salivary fluid was simulated by diluted commercial sialic acid powder in deionized water at 200 mg/DL with the addition of some specimens of silver (Ag) and gold (Au) nanoparticles (NPs) and dried at room temperature [101]. The samples were studied by the THz frequency-domain spectrometer in the range of 0.2 to 1.8 THz. Ag-NPs and Au-NPs slightly enhance the absorption at 1.32 THz, yet a dry nitrogen atmosphere is required to remove the air contribution. Time-domain THz spectroscopy analysis of saliva was held to diagnose oral lichen planus (OLP) [149], which is a premalignant condition in the oral cavity. The investigation included a total of 30 patients from two subgroups with 15 patients belonging to the erosive form of OLP and 15 from the reticular and papillar forms of OLP. Informative features were selected by the PCA and classified by one-vs-one multiclass SVM which provided 100% accuracy of separation between the classes. A very promising application of THz spectroscopy is microRNA detection in biofluids. MicroRNAs present a novel class of evolutionarily conserved small non-coding single-stranded RNA molecules of 18–22 nucleotides in length [102, 150]. It has been reported that several cancers can be diagnosed by the miR-200 family [143, 151]. Another cancer diagnostic agent found in saliva and blood is membrane-bound extracellular vesicles produced in the endosomal compartment – exosomes. A study of exosomes in biofluids by THz laser spectroscopy was carried out [152]. It examined 6 samples obtained from patients diagnosed with colorectal cancer, 4 samples from patients with colon polyps, 4 samples from healthy people, and 1 sample from a patient with colorectal precancer. The studied exosome samples were stored under standard conditions in a phosphate-salt solution (buffer liquid) at a temperature of − 80 °C. The samples were defrosted slowly before measurement. Measurements were carried out with the THz Time-Domain Spectroscopy device (EXPLA, Estonia). The intensity spectrum of the buffer liquid was used as a reference signal (as shown in Fig. 7a). Figure 7 b shows an example of optical density spectra for a healthy donor and patients with polyps and colorectal cancer.

**Fig. 8:**
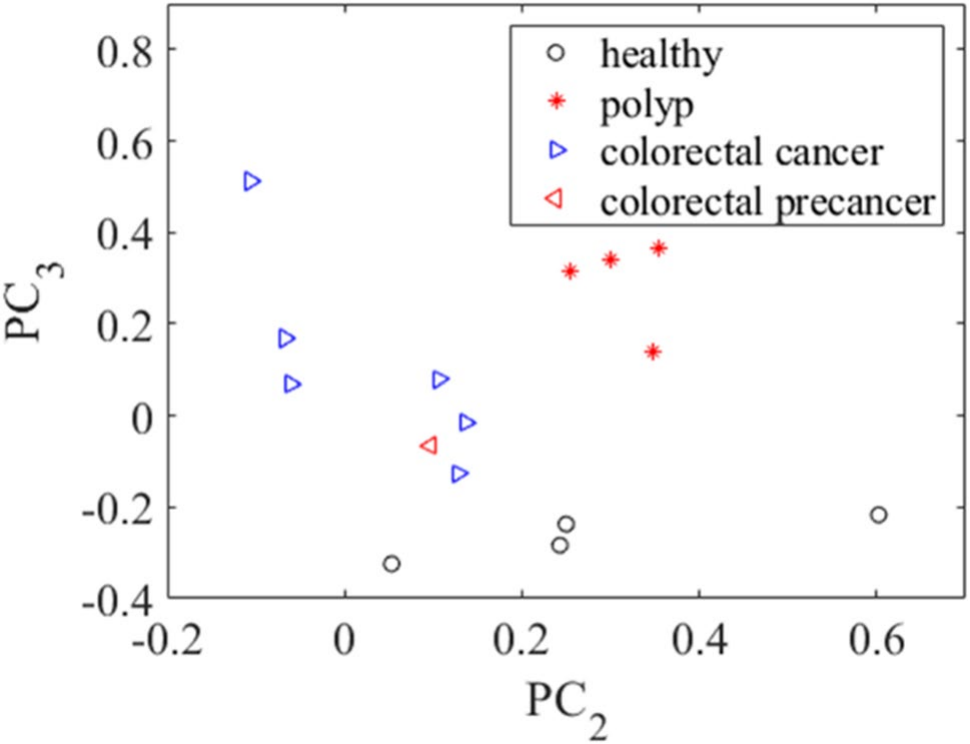
Projection of optical density spectra on the plane of the second and third principal components for exosome samples from the groups under study

Figure 8 shows the optical density THz spectrum projections on the plane of the second and third principal components. The spatial separation of exosome samples confirms this approach’s potential for colorectal cancer detection through exosome analysis by THz spectroscopy. Studies of blood plasma have been performed [153–155] using transmittance spectra in the 0.05–1.0 THz spectral range of the blood serum of rats with Ehrlich carcinoma were compared with healthy ones, and a decrease in the protein content was established. The same approach was applied to rats with implanted liver cancer cells. Besides differences in protein level, the biochemical composition of the blood serum also differed between healthy and cancer samples [103]. The presence of homocysteine in blood and urine is considered a risk factor for cancer [104, 156]. A dissolved homocysteine powder in ultrapure water was analyzed by Fourier transform infrared (FTIR) spectrometer with a spectral range of 0.9–20.0 THz [104]. The limit of concentration detection was about 10 µmol/L. Compared to Raman spectroscopy, THz spectroscopy has a higher correlation coefficient (0.80 versus 0.99).

## Conclusion

Optical methods are useful in characterizing and diagnosing different diseases. This review shows the importance of optical spectroscopy and microscopy techniques for detecting cancer. Fluorescence spectroscopy is used to distinguish normal mucosa and carcinoma of the lower gastrointestinal tract by measuring the autofluorescence signals from endogenous fluorophores for a set of excitation wavelengths. It is an example of the capability of the technique for determining healthy and diseased tissues. Fluorescence microscopy provides the spatial distribution of fluorophores with high contrast upon excitation of a particular wavelength. An SHG signal is generated from non-centrosymmetric molecules, including collagen, skeletal muscle, and microtubules present in cells and tissues. Raman spectroscopy, including CARS and SRS, can fingerprint the chemical bonds present in the sample without exogenous labeling. Photoacoustic spectroscopy and microscopy detect the acoustic signal with optical radiation. Again, in THz spectroscopy, the hydrogen bonds present in water molecules are highly absorbed by THz waves. The addition of the polarization component in these techniques will improve the image contrast and find new insight for tissue assessment. Again, the multimodal techniques have another potential for simultaneous measurement of several parameters in the same field of view.
